# Mycosynthesis of silver nanoparticles from endophytic *Aspergillus parasiticus* and their antibacterial activity against methicillin-resistant *Staphylococcus aureus in vitro* and *in vivo*

**DOI:** 10.3389/fmicb.2024.1483637

**Published:** 2024-11-15

**Authors:** Enas M. Ali, Peramaiyan Rajendran, Basem M. Abdallah

**Affiliations:** ^1^Department of Biological Sciences, College of Science, King Faisal University, Al-Ahsa, Saudi Arabia; ^2^Department of Botany and Microbiology, Faculty of Science, Cairo University, Cairo, Egypt; ^3^Centre of Molecular Medicine and Diagnostics (COMManD), Department of Biochemistry, Saveetha Dental College & Hospitals, Saveetha Institute of Medical and Technical Sciences, Saveetha University, Chennai, Tamil Nadu, India

**Keywords:** silver nanoparticles, MRSA, *Aspergillus parasiticus*, skin infection, wound healing

## Abstract

**Background:**

Methicillin-resistant *Staphylococcus aureus* (MRSA) is a drug-resistant and biofilm-forming pathogenic bacteria with severe morbidity and mortality. MRSA showed resistance against currently available antibiotics. Thus, there is an urgent need to develop novel effective treatments with minimal side effects to eliminate MRSA.

**Aim:**

In this study, we aimed to mycosynthesize silver nanoparticles (AgNPs) using the endophytic fungus *Aspergillus parasiticus* isolated from leaves of *Reseda Arabica* and to examine their antibacterial activity against MRSA.

**Results:**

Screening of fungal secondary metabolites using gas chromatography–mass spectroscopy (GC–MS) analysis revealed the presence of high content of bioactive compounds with antibacterial activities. AP-AgNPs were mycosynthesized for the first time using ethyl acetate extract of *A. parasiticus* and characterized by imaging (transmission electron microscopy (TEM), UV–Vis spectroscopy, zeta potential, X-ray diffraction (XRD), energy-dispersive X-ray analysis (EDX), and Fourier transform infrared spectroscopy (FTIR)). The agar well diffusion method revealed the antibacterial activity of AP-AgNPs against MRSA with 25 μg/mL of minimum inhibitory concentration (MIC). AP**-**AgNPs were shown to exert antibacterial action via a bactericidal mechanism based on flow cytometry, scanning electron microscopy, and transmission electron microscopy assessment. Our data demonstrated the effective interaction of AP-AgNPs with the bacterial cell membrane, which resulted in cell membrane damage and disruption of cell surface structure. Furthermore, AP-AgNPs successfully prevented the development of MRSA biofilms by disturbing cell adhesion and destructing mature biofilm reaching over 80% clearance rate. Interestingly, topical application of AP-AgNPs to superficial skin infection induced by MRSA in mice effectively promoted wound healing and suppressed bacterial burden.

**Conclusion:**

Our results provide a novel green nanoparticle drug design with effective therapeutic potential against MRSA-induced skin infection.

## 1 Introduction

Antibiotic resistance is recognized as the failure of antibiotics to produce effects on the growth of bacteria. Bacteria exhibit resistance to antibiotics via overuse or misuse of antibiotics. A number of cases of antibiotic resistance are growing, as observed in the substantial increase in morbidity and mortality rates resulting from infectious diseases. World Health Organization and the Center for Disease Control have confirmed that the disaster of antibiotic resistance is getting worse, as we are still living in the “post–antibiotic era” (Michael et al., 2014).

One of the actual severe and frequently emerging antibiotic-resistant bacteria is methicillin-resistant *Staphylococcus aureus* (MRSA) (Larsen et al., 2022). It is considered as the main cause of hospital-acquired infections, resulting in several diseases, for example, endocarditis, chronic osteomyelitis, pneumonia, septic arthritis, osteoarthritis, and bacteremia (Gao et al., 2021). MRSA has established multiple drug-resistant mechanisms to survive, including thickening of cell walls, increased efflux pumps, mutation of target drug, and biofilm formation (Singh et al., 2021). MRSA is resistant to several groups of antibiotics, including penicillin, linezolid, and daptomycin (Vestergaard et al., 2019). Furthermore, because of the development of drug resistance in bacteria and biofilm protection, accomplishing a reasonable therapeutic effect for bacteria-infected open wounds with conventional measures is problematic (Mirghani et al., 2022).

Owing to bacterial resistance, higher dosages of antibiotics are needed to produce a therapeutic action, often causing adversarial effects. For example, vancomycin, the primary antibiotic used in the treatment of MRSA, has several restrictions, including nephrotoxicity, lower tissue penetration, and lower antibacterial potential (Gao et al., 2021). Consequently, it is urgently essential to progress novel and operative anti-MRSA therapeutic approaches.

One approach that actually addresses these disadvantages is the use of metallic nanomaterials to evade MRSA antibiotic resistance by acting either as a drug delivery system and/or as effective antibacterial drugs, because of their broad-spectrum antibacterial activity. In this context, silver nanoparticles (AgNPs) are used effectively as an antibacterial agent to inhibit cell division and growth of multidrug-resistant pathogens including MRSA in several studies (Andrade et al., 2023). For example, AgNPs decorated with chlorin e6 (Ce6) and poly [4-O-(*α*-D-glucopyranosyl)-D-glucopyranose] (GP) showed significant antibacterial effects against MRSA and outstanding *in vivo* biocompatibility of the nanosystems (Xu et al., 2021). Synthesized gold–silver core–shell theranostic nanoparticles functionalized with an anti-MRSA antibody on their surface demonstrated high potential for the treatment of MRSA-induced pneumonia *in vitro* and *in vivo* (Huo et al., 2014). Interestingly, biosynthesized AgNPs using culture supernatants of *S. aureus* demonstrated high antimicrobial activities against different pathogenic bacteria including MRSA (Nanda and Saravanan, 2009). In addition, modified antibiotic-loaded AgNPs showed a high capacity to prevent MRSA growth *in vitro* (Huang et al., 2021).

Recently, the effectiveness of green methods for the synthesis of metallic nanoparticles has increased significantly. The advantages of green synthesis of AgNPs are lower toxicity, cost, and time as compared to chemical and physical approaches (Abada et al., 2024). Eco-friendly green synthesis of AgNPs using plant extracts is used efficiently against MRSA due to the presence of active phytochemicals with antimicrobial effects that act as capping agents of AgNPs. In this context, green synthesized AgNPs using the plant extract of *Foeniculum vulgare* or *Alysicarpus monilifer* leaf extracts showed significant antibacterial effect against MRSA *in vitro* and *in vivo* (Fatima et al., 2023; Kasithevar et al., 2017). Several microorganisms, such as fungi, bacteria, and yeast, were used to biosynthesize AgNPs via extracellular or intracellular pathways (Rudayni et al., 2022). Fungi such as *Aspergillus niger* and *Penicillium* sp. can produce large quantities of effective proteins and were being used successfully in the extracellular mycosynthesis of AgNPs (El-Zawawy et al., 2023; Sastry et al., 2003). However, endophytic fungi-based AgNPs have not yet been applied as a complementary and alternative antibacterial therapy against MRSA.

Endophytic fungi that colonize the internal tissues of plants in a mutualistic relationship are considered a potential source of bioactive metabolites with antimicrobial activities (Caruso et al., 2022; Gupta et al., 2023). Endophytic fungi-based biomolecules have been reported as complementary and alternative antibacterial therapy against pathogenic bacteria (Cadelis et al., 2021; Caruso et al., 2023). By description, every individual plant on earth is a host for one or more endophytes (Strobel and Daisy, 2003) and these endophytes spend all or part of their life inhabiting asymptomatically within the host plant tissues (Nair and Padmavathy, 2014). Several research studies have revealed that endophytes are significant sources of secondary metabolites with varied biological actions such as antiviral, anticancer, antioxidant, and antimicrobial properties (Khalil et al., 2021). Secondary metabolites such as phenols, alkaloids, saponins, steroids, terpenoids, flavonoids, and glycosides are accountable for these activities (Sharaf et al., 2022). Nevertheless, only 6–7% of the expected 1.5 million fungal species, including endophytes, are reported; therefore the rest are presently expecting inclusion in the known microbial world. Furthermore, 51% of the bioactive molecules detected from these endophytic fungi were formerly unknown (Hashem et al., 2023).

Plants used in traditional medicine are rich sources for exploring novel bioactive metabolites producing strains of endophytic fungi (Kaul et al., 2012). *Reseda* is a leading genus of the Resedaceae family that is extensively distributed in the Al-Ahsa Oasis (Al-Qurainy et al., 2022). *Reseda* species reported to have anti-inflammatory, antioxidant, antibacterial, and antimicrobial activities (Chibani et al., 2014; Shahat et al., 2017). The extracts of *Reseda muricata* are used in external treatments of hemorrhoids and for stomach aches and diarrhea in traditional medicine (El-Sayed et al., 2001). There is no chemical report on *R. arabica* in the literature.

Thus, this study aimed to mycosynthesize AP-AgNPs for the first time using the endophytic fungus *Aspergillus parasiticus* isolated from the leaves of *Reseda arabica* and investigate their antibacterial activity against MRSA *in vitro* and *in vivo* using MRSA-induced superficial skin infection mouse model.

## 2 Materials and methods

### 2.1 Isolation and identification of endophytic fungi

Fresh healthy leaves of *Reseda arabica* were collected from Al-Ahsa Oasis in Saudi Arabia’s Eastern region (25°20002.900 N 49°34039.900 E). The samples were washed with running tap water within 24 h of collection, then washed with double-distilled deionized water to remove surface debris. The cleaned leaves were allocated into 1 cm × 1 cm-size segments. Surface sterilization was carried out under aseptic conditions with succeeding washes in 70% ethanol, 4% sodium hypochlorite solution, sterilized distilled water, and finally allowed to air dry in the laminar airflow chamber (Dobranic et al., 2011). The sterilized leaf segments were added to potato dextrose agar (PDA) supplemented with 100 μg/mL streptomycin to prevent bacterial growth and incubated for 5–7 days at 28°C until endophytic fungi start to develop hyphal filaments. The growth was observed daily, and individual hypha tips were picked out on time and transferred to a new PDA medium until pure fungal colonies were obtained. Morphological identification of the isolated endophytic fungi was performed at the Regional Center for Mycology and Biotechnology, Al-Azhar University, Cairo, Egypt. All isolated endophytic fungi were screened for their antibacterial activities in addition to the ability of their extracts to reduce AgNO_3_ into AgNPs (data not shown). *A. parasiticus* EA-1 (RCMB 150) displayed significant activity against MRSA and powerful potential to reduce AgNO_3_ into AP-AgNPs. Therefore, it was further selected for the mycosynthesis of AP-AgNPs.

### 2.2 Genotypic identification

Sequence analysis of the ITS1-5.8S-ITS2 and ITS regions of the ribosomal ribonucleic acid (RNA) gene was performed to confirm the morphological identification of the *A. parasiticus* EA-1 (RCMB 150) isolate. Briefly, the total fungal genomic DNA was directly obtained from fungal mycelia. The isolated DNA was subjected to the polymerase chain reaction (PCR, Applied Biosystems, USA) using primers ITS1: TCCGTAGGTGAACCTGCGG and ITS4: TCCTCCGCTTGATATGC (White Bruns et al., 1990). Then, the BigDye Deoxy Terminator Cycle Sequencing Kit (Applied Biosystems, Darmstadt, Germany) was used to purify the amplified product, and the sequence was obtained by using an automated DNA sequencer (ABI PRISM 3700). The developed fungal sequence was compared with previous species sequences in NCBI GenBank. The identification of endophytic fungus was performed depending on the homology of amplified sequences to those in the GenBank database. The relevant accession number of isolate was obtained using MEGA version 7.0, and the phylogenetic analysis of sequences was also created.

### 2.3 Extraction of fungal active compounds

Fungal isolate was cultured on potato dextrose broth (PDB) medium and kept at 25°C for 20 days in a shaking (150 rpm) incubator and extracted by using ethyl acetate. Filtrate was mixed with an equal volume of ethyl acetate, placed on a vortex shaker for 15 min, and kept for 4 min until the two clear separate layers. The ethyl acetate layer was separated from the aqueous layer using a separating funnel. The collected phase was evaporated using the oven at 65°C. Finally, DMSO (1 mg/mL) was used to dissolve the fungal crude extract and then stored at −20°C until further experiments.

### 2.4 Screening of fungal secondary metabolites using gas chromatography–mass spectroscopy (GC–MS) analysis

Qualitative screening of fungal secondary metabolites (alkaloids, flavonoids, glycosides, steroids, terpenoids, tannins, saponins, and reducing sugars) was carried out following the methods of Gul et al. (2017).

The metabolites existing in the ethyl acetate extract of *A. parasiticus* were examined, counted, and identified using GC–MS. Briefly, the fungal extract was dissolved in spectroscopy-grade methanol. GC–MS analysis was done using Trace GC1310ISQ mass spectrometer (Thermo Scientific, Austin, TX, USA). The column oven temperature was 50°C at the start and increased at a rate of 5°C/min to 230°C and then held for 3 min, and then raised to the final temperature of 290°C and kept for 3 min. The sample (1 μL) was injected at 250°C utilizing helium as a carrier gas, split at the ratio of 1:30. Mass spectrometer functioned in the electron ionization mode at 200°C at 70 eV with a scan range of 40–1,000 m/z. The spectrum of the identified compounds was compared with the spectrum of the known compounds stored in the WILEY 09 (Wiley, New York, NY, USA) and NIST 11 (National Institute of Standards and Technology, Gaithersburg, MD, USA) library.

### 2.5 Mycosynthesis and characterization of AP-AgNPs

Ethyl acetate extract of *A. parasiticus* was obtained as described previously, and 200 μL of 0.5 M AgNO3 solution was added to 100 mL of fungal extract to obtain a final concentration of 1 mM AgNO3. The mixture was incubated at 25°C; 150 rpm for 2 days in the dark. Then, the mixture was checked for brown color, which indicated the formation of AP-AgNPs. The particles were harvested by ultracentrifugation at 5°C, 15,000 rpm for 10 min, and freeze-dried using benchtop lyophilizer. A schematic diagram of the mycosynthesis mechanism of AP-AgNPs is displayed in Supplementary Figure S1B.

### 2.6 Characterization of mycosynthesized AP-AgNPs

The biosynthesis of AP-AgNPs was confirmed by recording the absorption spectra using UV–Vis spectroscopy at a wavelength of 300–700 nm (Elico UV–visible double-beam spectrophotometer). X-ray diffraction (XRD) analysis was performed by XPERT-PRO using monochromatic Cu-Ka radiation (k = 1.5406 A°) functioned at 40 kV and 30 mA at a 2 h angle design. The scanning was done in the region of 208–808. The images obtained were compared with the Joint Committee on Powder Diffraction Standards (JCPDS) library to explain the crystalline structure. Fourier transform infrared spectroscopy (FTIR) is used to identify the functional groups of biological molecules accountable for the biosynthesis and capping of AP-AgNPs. Potassium bromide was added to the dried AP-AgNP powder (100:1), and the FTIR spectrum was recorded using an FTIR Spectrophotometer (PerkinElmer) in the range of 450–4,000 cm^−1^. TEM (TEM HF-3300—Hitachi High-Tech Canada, Inc.) is used to describe the shape and size of AP-AgNPs. The size distributions from the TEM images for AP-AgNPs were subsequently examined using ImageJ Freeware Version 1.53d downloaded from the NIH website.^1^ The zeta potential of the biosynthesized AP-AgNPs was determined by a nanoparticle analyzer (Zetasizer Ver. 7.13, Malvern Instruments Ltd., UK). Energy-dispersive X-ray spectroscopy (EDX) at 20 keV was used to confirm the occurrence of nano-silver elements.

### 2.7 Bacterial strain

The bacteria used in this study were methicillin-resistant *staphylococcus aureus* (MRSA) ACLT 32571 clinical isolate obtained from Kasr Al-Aini Hospital, Cairo University, Cairo, Egypt.

### 2.8 Antibacterial susceptibility test

#### 2.8.1 Disk diffusion method

Pure isolate of MRSA subcultured on the agar media plates at 37°C for 24 h. One plate was taken, and a minimum of three colonies were touched with a sterile loop and transferred to normal saline (0.80%) under aseptic conditions. The density of bacterial suspension was standardized to 10^7^ CFU/mL and used as the inoculum for agar well diffusion assay. One hundred microliters of inoculum of MRSA was spread onto the agar plates, and the plates were allowed to dry. Wells of 6 mm were made in agar plates, and the lower portion of each well was closed with a little specific molten agar medium. The AP-AgNPs were reconstituted in 20% DMSO for the analysis (Chandrasekaran et al., 2008). A 100 μL volume of AP-AgNPs was propelled directly into the wells (in triplicates) of the inoculated agar plates for the test organism. The plates were allowed to stand for 1 h for diffusion of the antifungal agent into the agar and incubated at 37°C for 24 h. Sterile DMSO (20%) was used as the negative control and vancomycin served as the positive control.

#### 2.8.2 Determination of minimum inhibitory concentration (MIC)

A 2-fold serial dilution of AP-AgNPs, vancomycin, and DMSO was prepared by first reconstituting the compound in 20% DMSO followed by dilution in sterile distilled water (1:1) to achieve a decreasing concentration range of 200 μg/mL to 6.25 μg/mL. A 100 μL volume of each dilution was introduced into wells (in triplicate) in the agar plates already seeded with 100 μL of standardized inoculum (10^7^ CFU/mL) of the MRSA. All test plates were incubated aerobically at 37°C for 24 h, observed for the inhibition zones, and MIC was determined as described (Nkere and Iroegbu, 2005).

#### 2.8.3 Time–kill assay

One milliliter of AP-AgNPs or vancomycin was added to 29 mL of MRSA bacterial suspension, at concentrations of 25 and 50 μg/mL (MIC), for AP-AgNPs and vancomycin, respectively. The bacterial density was adjusted to 10^9^ CFU/mL. DMSO was set as the control. For viable counts examination, 1 mL of sample was taken from the inoculated solutions every 2 h and then serially diluted to 10-fold with 0.85% saline and spot-plated in triplicate. These plates were incubated at 37°C for 24 h (Lorian, 2005).

### 2.9 Scanning electron microscopy (SEM)

MRSA suspension was fixed by 1% glutaraldehyde and then dehydrated in alcohol solutions (30, 50, 70, 80, 90, and 100%) and allowed to air dry and finally coated with Au/Pd (Quorum Q150T ES vacuum coater). Pictures were acquired by the SEM Merlin (Carl Zeiss, Jena, Germany), working at an accelerating voltage of 15 kV, SE-detector.

### 2.10 Membrane permeability

The effect of AP-AgNPs on membrane permeability was assessed as described (Marri et al., 1996). 10 μL of 1x AP-AgNPs, 10 μL of ortho-nitrophenyl-galactoside (ONPG), and 30 μL of the bacterial suspension were mixed in triplicate in the wells of a 96-well microtiter plate. Triton X-100 was used as the positive control. Samples were incubated at 37°C for 3 h, and the OD was measured every 30 min at 405 nm.

### 2.11 Hemolytic activity

The red blood cells were counted and diluted to a concentration of 10^7^–10^8^ cells/ml and incubated at room temperature for 1 h with serially diluted AP-AgNPs at a ratio of 4:1 in a final volume of 100 μL. The optical density at 570 nm was measured with a microplate reader. HC_50_ values were obtained following the method of Cao et al. (2012). Saline solution at a concentration of 0.85% was used as the negative control, and 0.1% Triton X-100 was used as the positive control.

### 2.12 Assessment of intracellular constitutes leakage

The leakage of intracellular proteins and nucleic acids was assessed as described (Moghimi et al., 2016). Briefly, the bacterial suspension was centrifuged at 7000 rpm for 4 min, filtrated, and examined at the absorption of 260 nm and 280 nm using an ultraviolet spectrophotometer (Agilent, USA).

### 2.13 Transmission electron microscopy (TEM)

MRSA cells were cultured in Brain Heart Infusion (BHI) culture medium for 24 h at 37°C and were centrifuged at 3000 rpm. Subsequently, cells were washed with PBS buffer and fixed overnight in 2.5% glutaraldehyde and 4% paraformaldehyde in 0.1 M cacodylate buffer, with pH 7.2. Then, the cells were fixed for 30 min in 1% OsO4, with dehydration in acetone, and embedded in a solution of Polybed 812. Finally, ultra-thin sections were selected on 300-mesh copper grids and stained with uranyl acetate (5%) and lead citrate (1%). The images were photographed with an FEI Tecnai G2 Spirit transmission electron microscope, working at 120 kV.

### 2.14 Confocal laser scanning microscopy (CLSM)

The bacterial cell survival was assessed after 24 h of treatment with AP-AgNPs and observed by CLMS on an inverted Carl Zeiss LSM 780 confocal laser scanning microscope (Carl Zeiss, Jena, Germany). The samples were stained for 20 min with acridine orange (green fluorescent) and propidium iodide (red fluorescent) to distinguish between undamaged and cell surface damaged bacteria.

### 2.15 Evaluation of cell membrane integrity

PI staining using flow cytometry was used to determine the cell membrane integrity following the method of Ning et al. (2017). The MRSA suspensions were washed with PBS, and the bacterial precipitate was re-suspended by adding 100 μL 1× binding buffer. Subsequently, 10 μL PI staining solution was added to the suspensions and mixed gently. After 10–15 min in darkness at room temperature, samples were measured by flow cytometry (FACS CantoI1, USA) in 1 h.

### 2.16 Inhibition of biofilm formation

MRSA cells (1.5 × 10^8^ CFU/mL) were mixed with AP-AgNPs (25 to 200 μg/mL) in LB broth in 96-well plates, and cells were incubated for 24 h at 37°C. After incubation, each well was rinsed three times with sterile 0.85% saline by discarding the culture supernatant, and then, the adherent biofilm was fixed with methanol and stained with 0.2% crystal violet (7 min). Cell growth was determined by measuring the OD at 595 nm (Shi et al., 2023). AP-AgNPs mixed in polystyrene 24-well plates with bacterial suspensions (1.0 × 10^6^ CFU/mL) at different concentrations in LB broth. After 2 days of incubation, wells were washed with a sterile 0.85% saline, and a 0.01% final concentration of resazurin was added, followed by an incubation in the dark for 1 h. A microplate reader (Thermo Fisher Scientific, Waltham, MA, USA) was used to measure the optical density (OD 570 and OD 600) values (Paduszynska et al., 2019). Furthermore, we performed additional experiments on the inhibitory potential of AP-AgNPs in the establishment of MRSA biofilms following the method proposed by Hu et al. (2024). Briefly, pictures of biofilms were taken for control cells and for cells treated with AP-AgNPs at concentrations of 25 μg/mL (MIC), 50 μg/mL, or 100 μg/mL; 2 mL of MRSA-diluted overnight culture was used to grow biofilms on coverslips for 48 h. The cover slides were then washed with PBS, transferred to a new plate, and exposed to AP-AgNPs for 48 h. The cover slides were washed again with PBS and stained with a LIVE/DEAD BacLight Bacterial Viability Kit (Invitrogen Molecular Probes, Eugene, OR, USA). CLSM pictures were taken using an Olympus FV1000 confocal laser scanning microscope (Olympus, Tokyo, Japan) with a 60× objective lens. For the examination of SYTO 9 (green channel), we used 488 nm excitation and 520 nm emission filter settings. For PI detection (red channel), we used 543 nm excitation and 572 nm emission filter settings. Picture investigations were carried out using Fluoview version 1.7.3.0 software.

### 2.17 Mouse superficial skin infection model

The *in vivo* Tape-Stripping Mouse Model was approved and licensed by the Research Ethics Committee, Deanship of Scientific Research at King Faisal University (KFU-REC-2022-OCT-ETHICS218). Male BALB/c mice (*n* = 24, 2 months old) were housed under conventional conditions of 25 ± 2°C and 12 h dark/light cycle, with a standard diet and water *ad libitum*.

The mice were anesthetized by a combination of 100 mg/mL ketamine and 20 mg/mL xylazine, and the back hair was shaved. Small pieces of elastic bandage tape were applied and immediately removed from a 2 × 2 cm^2^ dorsal area for 10 to 15 times until the skin became enlarged and ruddy without bleeding. Subsequently, a 10 μL-MRSA inoculum (1 × 108 CFU/mL) was applied to wounds (Tatiya-Aphiradee et al., 2016). Mice were divided into four groups (*n* = 6 per group) as follows: Group 1, non-infected and non-treated (negative control group). Group 2, MRSA-infected group with saline. Group 3, MRSA-infected group treated topically to the wound area with a 100-μL aliquot of 10% ethanol in propylene glycol (vehicle) and 160 μg/mL of vancomycin daily for 9 successive days (as a positive control group). Group 4, the MRSA-infected group treated topically with a 100-μL aliquot of each sample (10% ethanol in propylene glycol) (vehicle), 100 μg/mL of AP-AgNPs daily for 9 successive days. Wounds photographed on days 2, 4, 6, and 8.

#### 2.17.1 Bacterial burden

To determine the antibacterial effect of AP-AgNPs, the number of MRSA colonies was recorded during the wound healing. Wounds were swabbed every 2 days, and MRSA colony counts were performed on the MSA plates after incubation for 18–24 h (Tatiya-Aphiradee et al., 2019).

#### 2.17.2 Histology analysis

Mice were killed on days 2, 4, and 8. The skin from the dorsal area was collected and washed in phosphate-buffered saline for 20 min before soaking in a 15% formalin solution at 4°C for 24 h. Samples gradually dehydrated in ethanol (50 to 100% v/v) at room temperature, embedded in warm paraffin (60°C), and frozen at −20°C. The paraffin-embedded skin was cut into 5 μm sections, fixed on a glass slide, and stained with hematoxylin and eosin (H&E) solution and Masson’s trichrome (Tawfik et al., 2016).

### 2.18 C-reactive protein measurement

C-reactive protein (CRP) was evaluated with enzyme-linked immunosorbent test (ELISA) kits (R&D Systems, Kamiya Biomedical Company), following the manufacturer’s instructions.

### 2.19 Statistical analysis

All values expressed as mean ± SD (standard deviation) of at least three independent experiments. Power calculation was performed for using unpaired two-samples Student’s *t*-test (two-tailed) assuming equal variation in the two groups. Differences were considered statistically significant at *p < 0.05 and **p < 0.005.

## 3 Results

### 3.1 Molecular identification of endophytic fungus

Genotypic methods and morphological characterization were used to verify the identification of the most promising endophytic fungal isolate, EA-1 (RCMB 150), *A. parasiticus*. Depending on the partial sequence of the 18S ribosomal RNA gene; internal transcribed spacer 1, 5.8S ribosomal RNA gene, and internal transcribed spacer 2; the complete sequence and the partial sequence of 28S ribosomal RNA gene, the fungal sequence was submitted to NCBI GenBank database with accession number PQ460365. Higher similarity sequences were selected, MEGA 7.0 was used to construct a phylogenetic tree depending on the maximum probability method (Supplementary Figure S1A), and the isolate was closely related to a cluster of *A. parasiticus* according to BLAST analyses.

### 3.2 Screening the fungal secondary metabolites of *Aspergillus parasiticus* extract using GC–MS analysis

**Figure 1:**
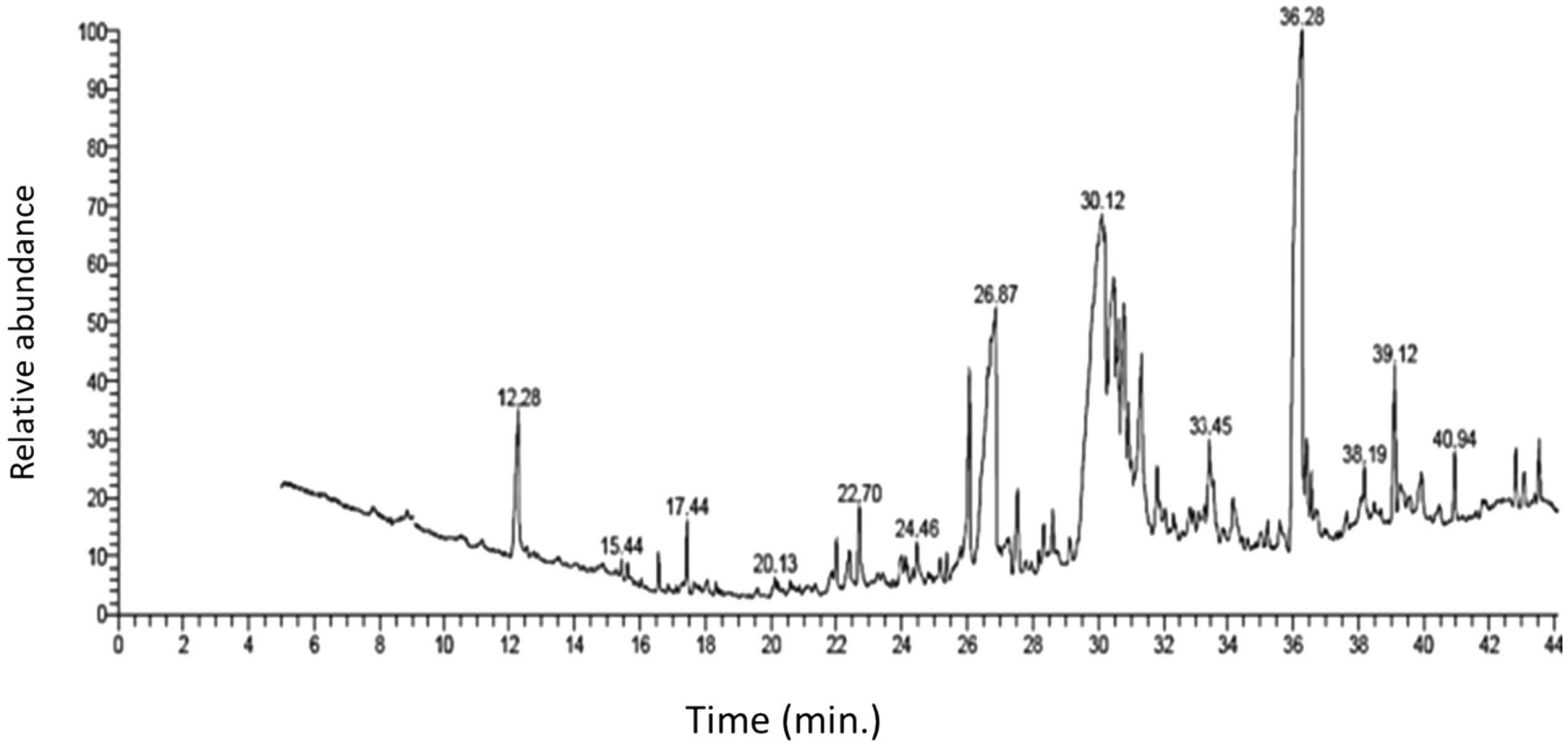
GC–MS chromatogram of ethyl acetate extract of *A. parasiticus.*

To identify the fungal secondary metabolites with antibacterial activity in *A. parasiticus* extract, we performed GC–MS analysis. The results of the screening are given in Table 1, which revealed some secondary metabolites that are present in the ethyl acetate extract of *A. parasiticus*. GC–MS analysis showed a demonstrative spectral output of each one of the compounds present in the examined samples. The results from the analysis of ethyl acetate extract of *A. parasiticus* determined that the average yield of the extract is approximately 16 major compounds as shown in Figure 1 and Table 2. 1,2-Benzenedicarboxylic acid, di-iso-octyl ester; 9-octadecenoic acid, E; and hexadecanoic acid were the major compounds with ratios 25.88, 16.93, and 8.31%, respectively.

**Table 1:** Phytochemical screening of ethyl acetate extract of *A. parasiticus*.

No	Secondary metabolite	Extract
1	Alkaloids	−
2	Glycosides	+
3	Flavonoids	+
4	Steroids	−
5	Terpenoids	+
6	Tannins	−
7	Reducing sugars	−
8	Saponins	−

**Table 2:** Chemical compounds and their retention time (min) identified in the ethyl acetate crude extract of *A. parasiticus* using GC–MS.

No.	Compound name	Retention time (min.)	Peak area (%)
1	Benzaldehyde, 4-nitro-	12.28	2.64
2	1,2-Dihydro-4-methyl-6-nitro-2-oxo quinoline	22.7	1.35
3	1,2-Benzenedicarboxylic acid, butyl decyl ester	26.07	2.93
4	Hexadecanoic acid	26.86	8.31
5	2 h-Pyran, tetrahydro-2-(12-penta decynyloxy)-	27.53	1.55
6	9-Octadecenoic acid (Z)-, methyl ester	28.61	2.18
7	9-Octadecenoic acid (E)-	30.12	16.93
8	10,13-Octadecadiynoic acid, methyl ester	30.61	3.95
9	Androstan-17-one, 3-ethyl-3-hydroxy-,	30.79	3.66
10	9,12-Octadecadienoic acid (Z,Z)	31.32	2.77
11	Androst-4-en-3-one, 17-methoxy-, 3-methoxime (17á)-	32.8	1.53
12	Eicosanoic acid	33.45	2.31
13	1,2-Benzenedicarboxylic acid, diisooctyl ester	36.26	25.88
14	Pyridine, 2,4,6-triphenyl-	39.12	2.67
15	Ethyl iso-allocholate	39.92	2.39
16	Isochiapin B	40.94	5.15

### 3.3 Mycosynthesis and characterization of AP-AgNPs

**Figure 2:**
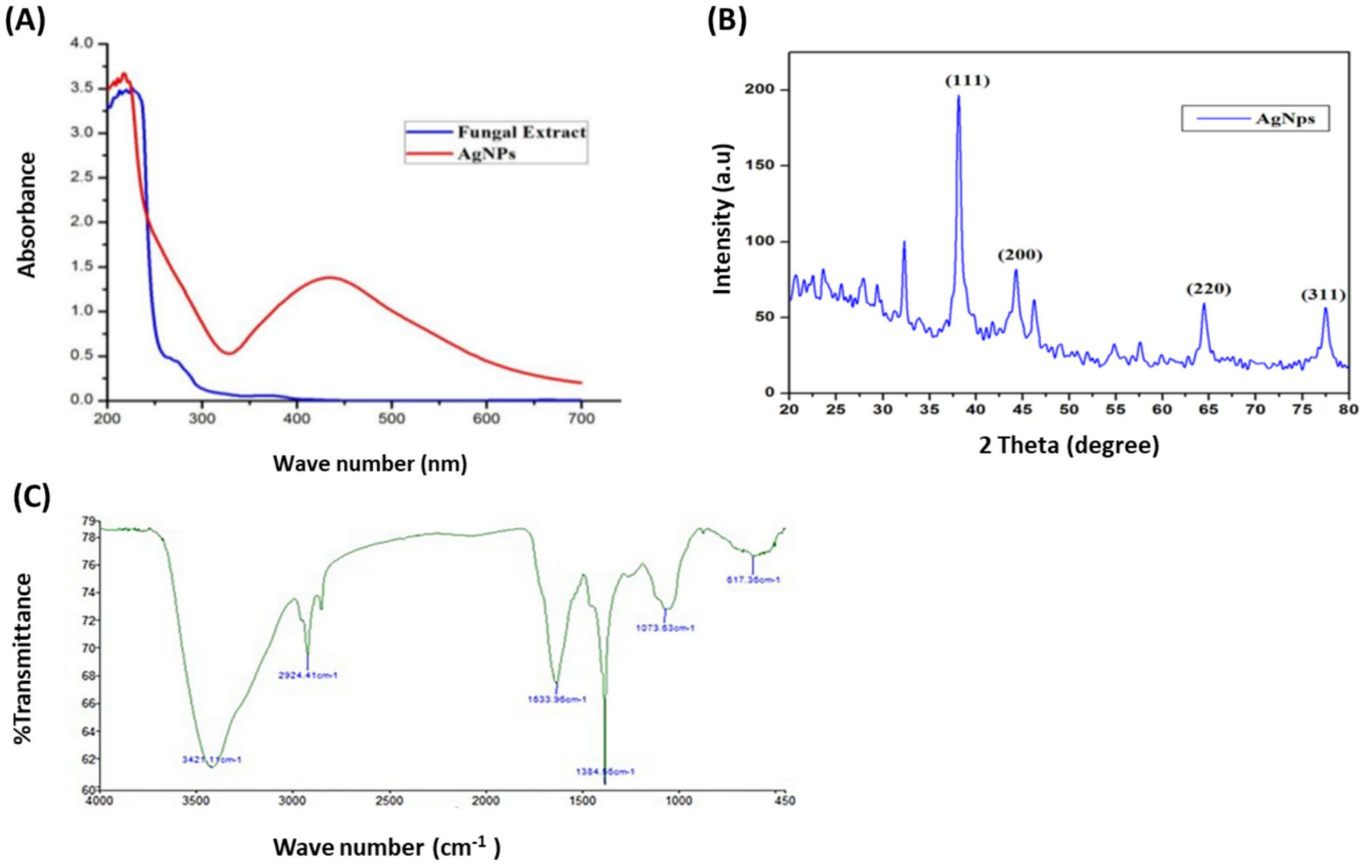
Confirmation of biosynthesized silver nanoparticles (AP-AgNPs). (A) UV–Vis spectrum of fungal filtrate and AP-AgNPs that were synthesized by the *A. parasiticus*. The peak values are for the UV–Vis plotted between AP-AgNPs and absorbance ratios. The highest absorbance peak of AP-AgNPs was at approximately 448 nm, corresponding to the Plasmon resonance of AP-AgNPs. (B) XRD spectrum recorded for AP-AgNPs showed four distinct diffraction peaks at 38.19°, 44.37°, 64.56°, and 77.47° indexed 2-theta (degree) values of (111), (200), (220), and (311) crystalline planes of cubic Ag. (C) FTIR spectrum of silver nanoparticles synthesized by *A. parasiticus* showed peaks at 3,421.1, 2,924.41, 1,633.96, 1,384.56, 1,073.63, and 617.36 cm^−1^. Those peaks were, respectively, attributed to N-H stretching of primary amine of the protein, alkane C-H stretching, the stretching of conjugated alkane C=C, methylene tails of the protein (CH3-R), C-N of aliphatic amines of polyphenols, and O-H stretching.

*Aspergillus parasiticus*-based AgNPs (AP-AgNPs) were mycosynthesized using ethyl acetate extract of the endophytic fungus *A. parasiticus* isolated from the leaves of *Reseda arabica* plant. The color of the reaction mixture (mycelial extract and AgNO3) was detected at regular intervals, which changed from white to yellowish brown after 48 h (Supplementary Figure S1B). This color change is attributed to the process of excitation of surface Plasmon resonance within the biologically produced AP-AgNPs. The biosynthesis of AP-AgNPs in the aqueous solution was identified by recording the absorption spectra using UV–Vis spectroscopy. The maximum peak of fungus-mediated AP-AgNPs biosynthesis was observed at 434 nm (Figure 2A). The XRD pattern obviously displays the major peaks at (2-theta) 38.19, 44.37, 64.56, and 77.47 corresponding to the (111), (200), (220), and (311) planes, respectively (Figure 2B). In addition, two unassigned peaks were found at 32.25° and 46.21°. Unpredicted crystalline structures (32.25° and 46.21°) are also present.

**Figure 3:**
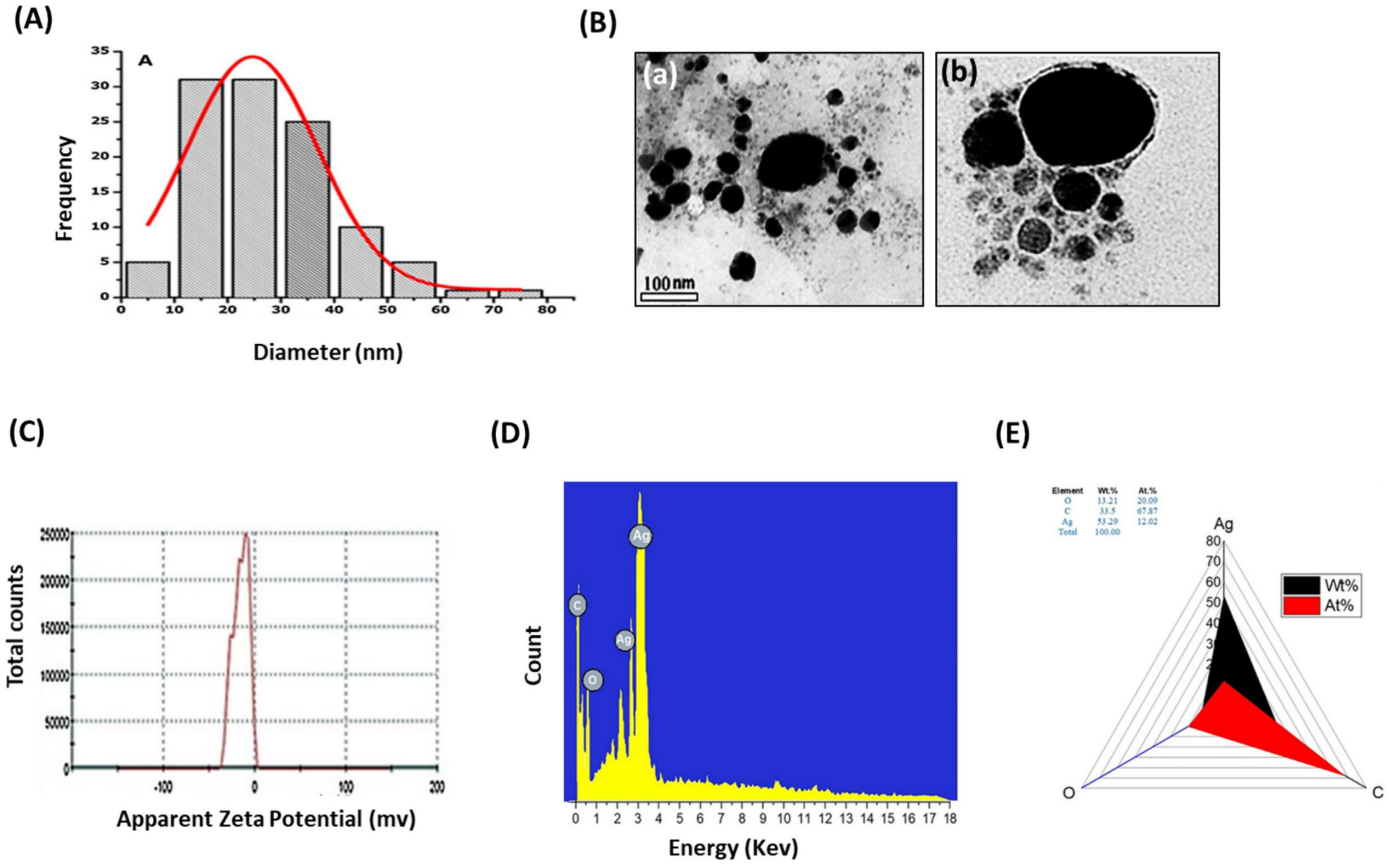
Characterization of AP-AgNPs. (A) Histogram of the particle diameter size distribution of the AP-AgNPs based on TEM image analysis. Red line: Gaussian distribution fit. (B) [a] Transmission electron microscopy (TEM) images of AP-AgNPs (scale bar = 100 nm). [b] High-resolution TEM image of nanoparticle of AP-AgNPs (scale bar = 20 nm). According to TEM, AP-AgNPs are mostly spherical, and the average particle size is 27.7 nm. The diameter range is 5.77–73.14 nm. The geometric mean equal value of 24.96 ± 1.65 nm. (C) Zeta potential of AP-AgNPs. The zeta potential value was −25.5 ± 3.15 mv. The negative value confirmed the stability of AP-AgNPs. (D) EDX images and (E) weight percentage of elements. The spectrum displayed that silver, constituting 53.29% of the total, was the greatest important element, followed by carbon (33.5%) and oxygen (13.21%), representing the establishment of AP-AgNPs.

The FTIR examination of AP-AgNPs showed strong peaks at 3421.1, 2924.41, 1633.96, 1384.56, 1073.63, and 617.36 cm^−1^ (Figure 2C). These peaks were equivalent to N-H stretching of primary amine of the protein, alkane C-H stretching, the stretching of conjugated alkane C=C, methylene tails of the protein, C-N of aliphatic amines of polyphenols, and O-H stretching, respectively. TEM examination of the size of AP-AgNPs revealed that the shape of AP-AgNPs is spherical, and the average particle size is 27.7 nm. The diameter range is 5.34–72.12 nm, and the geometric mean equal value is 24.93 ± 1.60 nm (Figures 3A,B). The stability of AP-AgNPs was assessed by zeta potential (Figure 3C). The zeta potential value was negative (− 25.4 ± 3.13 Mv), suggesting the good colloidal nature of biosynthesized nanoparticles. Energy-dispersive spectroscopy (EDS) was applied to obtain qualitatively evaluations of chemical compositions in nanoparticle samples; however, it is able to provide semi-quantitative results. The EXD (or EDS) results in Figure 3D showed variable intensities of signals at the X-axis of the several binding energies (keV), confirming the presence of diverse elements in the sample. A strong signal at 3 keV showed the occurrence of metallic silver in AP-AgNPs. Other weak-to-moderate signals were found in addition to the above-mentioned signals. These comprised the occurrence of oxygen and carbon because of the preparation of the sample using a copper grid coated with carbon in addition to the occurrence of carbon due to capping biomolecules. Figure 3E of the EDX spectrum displayed the elemental composition present except AP-AgNPs in the sample. The spectrum displayed that silver, constituting 53.29% of the total, was the greatest important element, followed by carbon (33.5%) and oxygen (13.21%), representing the establishment of AP-AgNPs.

### 3.4 Anti-MRSA activity of AP-AgNPs

**Figure 4:**
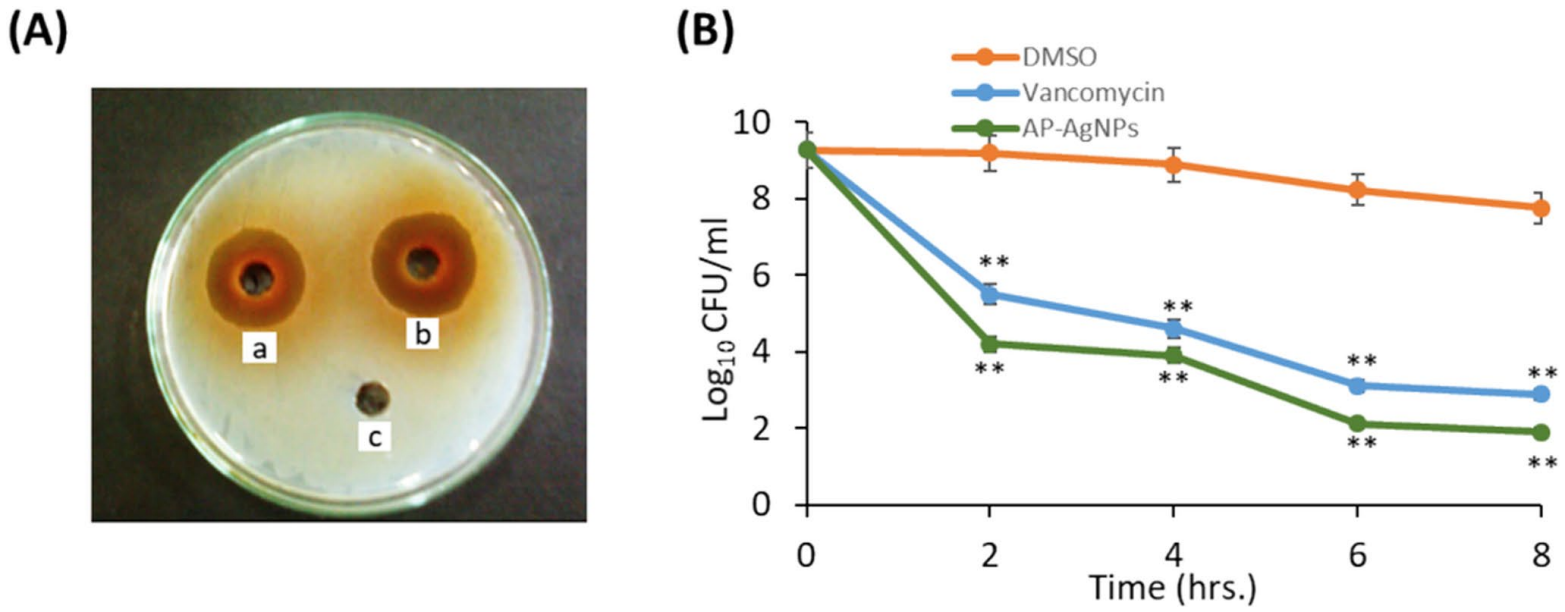
Antibacterial potential of AP-AgNPs. (A) Agar well diffusion method showing the antibacterial activity depicting the zone of antibacterial inhibition against MRSA by vancomycin (positive control) (50 μg/mL) (a), AP-AgNPs (25 μg/mL) (b), and negative control DMSO (c). Data are expressed as the mean zone of inhibition in millimeters. (B) Time–kill curves of MRSA following exposure to AP-AgNPs, vancomycin, and DMSO. Non-treated MRSA cells were used as a control. There was a decrease in the rate of cell growth when MRSA cells were treated with AP-AgNPs compared to the control cells. Values are means ± SD (**p < 0.005, compared to control DMSO-treated cells).

We examined the anti-MRSA activity of AP-AgNPs using the agar well diffusion method. The AP-AgNPs showed higher anti-MRSA action as compared to vancomycin where the diameter of the inhibition zone (IZD) was 48 and 42 mm, respectively (Table 3; Figure 4A). The MIC values of AP-AgNPs and vancomycin were 25 and 50 μg/mL, respectively (Table 3). The experiments of killing kinetics established there was a significant reduction in cell quantity after adding AP-AgNPs for several hours. Precisely, the curve of killing kinetics in Figure 4B revealed that it would take 2 h at least to recognize 1 log decrease in live cells, confirming the higher efficacy of AP-AgNPs as a potent anti-MRSA agent.

**Table 3:** Antibacterial activity of AP-AgNPs against MRSA.

Concentration (μg/mL)	Antibacterial agent		
	DMSO	Vancomycin	AP-AgNPs
	IZD (mm)		
0	_a_0^a^ ± 0.0	_a_0^a^ ± 0.0	_a_0^a^ ± 0.0
6.25	_a_0^a^ ± 0.0	_b_15^b^ ± 0.7	_b_33^c^ ± 0.7
12.5	_a_0^a^ ± 0.0	_c_22^b^ ± 0.5	_c_41^c^ ± 0.5
25	_a_0^a^ ± 0.0	_d_33^b^ ± 0.5	_d_48^c^ ± 0.8
50	_a_0^a^ ± 0.0	_e_42^b^ ± 0.5	No growth
100	_a_0^a^ ± 0.0	No growth	
200	_a_0^a^ ± 0.0		

IZD, Inhibition zone diameter (mm). Data are expressed as the mean zone of inhibition in mm followed by SD. The values with different subscript letters in the same column and those with different superscript letters in the same row are significantly different according to ANOVA and Duncan’s multiple range tests.

### 3.5 Effect of AP-AgNPs on MRSA ultrastructure modification

**Figure 5:**
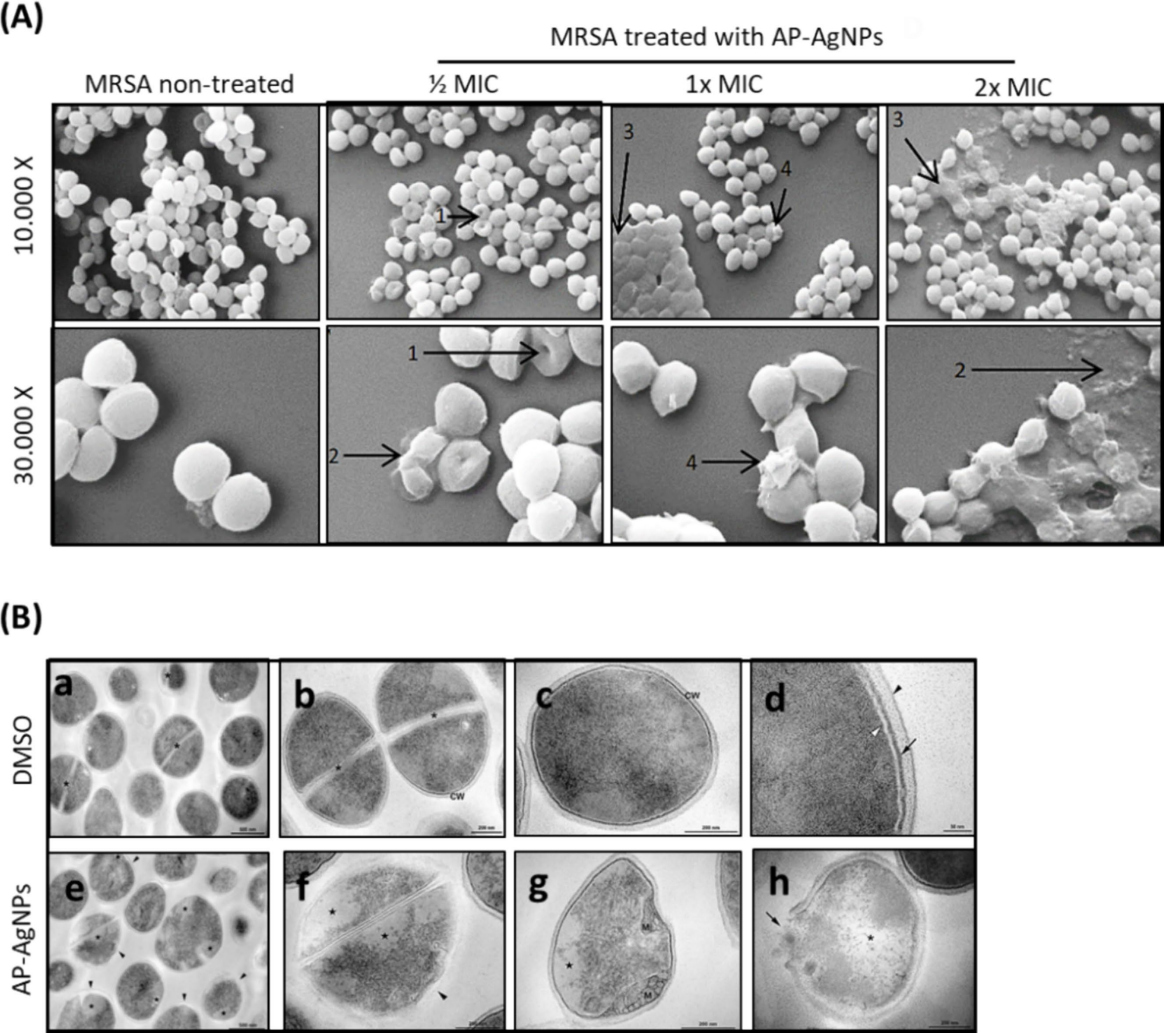
Effect of AP-AgNPs on MRSA ultrastructure. (A) Scanning electron microscopy of MRSA cells after 24 h of exposure to AP-AgNPs in nutrient broth. The cleavage sites (1), cellular debris (2), boss-like protuberances (3), and unusual protrusions (4) are shown by arrows. (B) Transmission electron microscopy of MRSA. (a–d) showing cells treated with DMSO (control). (a) General view displays rounded cells with a thick cell wall envelope and homogeneous electron density in the cytoplasm. Septa (*) appears in some cells. (b,c) Detailed view of non-dividing (b) and dividing (c) cells. A tripartite cell wall (CW) is seen enclosing the plasma membrane. (d) Inset of the cell wall. The black arrowhead specifies the outer highly stained fibrous surface and intermediate translucent region; arrow points to a heavily stained inner thin zone; the plasma membrane (white arrowhead) is seen below this electron-dense layer of the wall. (e–f) Showing cells treated with AP-AgNPs (MIC = 25 μg/mL) (M/4). (e) General view: some cells showed abnormal electron density in the cytoplasm and altered cell wall with no visible tripartite layers (black arrowheads). (f) Inset of figure e. (g) Detailed view of a cell showing alterations in the shape, loss of cytosolic electron density, and mesosome-like structures (M). (h) A lysed cell with cell wall disruption (arrow) and cytoplasmic disintegration (*).

The morphological alterations of cellular membranes of MRSA treated with AP-AgNPs for 24 h were examined by scanning electron microscopy (SEM). The untreated cells had intact, smooth, and spherical morphology (Figure 5A). The MRSA cells treated with 12.5 μg/mL (1/2 MIC) of AP-AgNPs displayed a moderate rise in the number of lysed cells. The treatment with higher concentrations of AP-AgNPs (25 and 50 μg/mL) resulted in a roughened bacterial surface with boss-like protuberances and unusual protrusions that were not detected in the control cells. In addition, these cells showed severely changed surfaces and noticeable cleavage sites (Figure 5A). Several lysed cells complemented by cellular debris and discharge of intracellular components could be perceived.

As shown in Figure 5B, non-treated MRSA displayed homogeneous cell walls without any obvious modifications and with complete cytoplasm as assessed using transmission electronic microscopy (TEM). In addition, non-treated MRSA showed the distinctive structures of *Staphylococci* morphology: rounded cells with a completely thick cell wall envelope and distinct membranes (Figures 5B,a–d). The cytosol showed a consistent electron density and proliferating cells with a central septum. The cell wall presented a tripartite structure consisting of an outer that displays an extremely stained fibrous surface, an intermediate transparent region, and an electrodense inner thin zone. The plasma membrane was detected immediately below this electrodense layer of the wall (Figures 5B,a–d). On the other hand, despite MRSA treated with AP-AgNPs showing rounded morphology and consistent electron density in the cytosol (Figures 5B,e–h), some cells presented modifications in the cell wall: without a tripartite-layers structure and no noticeable electrodense inner thin zone. In these cells, the observation of the plasma membrane was easier, rather than those under control conditions. Alterations in the outer extremely stained fibrous surface and intermediate transparent area of the cell wall were also detected.

### 3.6 Effect of AP-AgNPs on MRSA membrane integrity

**Figure 6:**
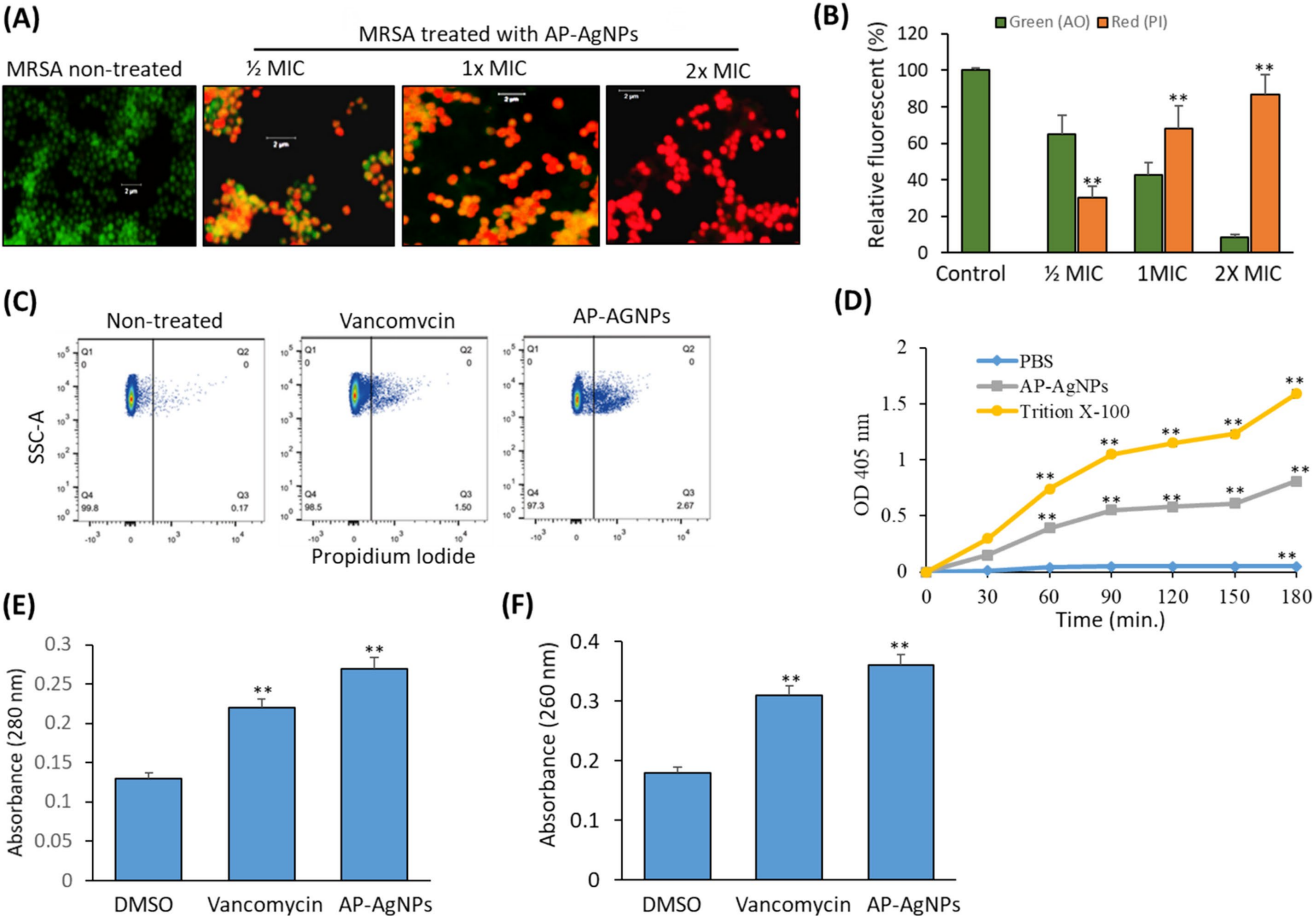
Effect of AP-AgNPs on MRSA cell membrane. (A) Fluorescent confocal microscopy of live and dead bacteria MRSA after 24 h of exposure to AP-AgNPs in nutrient broth and stained with propidium iodide (red color for dead bacteria) and acridine orange (green color for live bacteria). (B) Relative fluorescence intensity of PI and AO at different treatments. (C) Analysis of cell membrane integrity of MRSA by FACS analysis using PI staining under different treatments. (D) Effects of AP-AgNPs on the permeability of the cell membrane of MRSA. The membrane permeability was determined by the optical density value. AP-AgNPs and Triton X-100 increased the membrane permeability of MRSA, as compared with PBS (*p* < 0.01). (E) Leakage of intracellular proteins. (F) Leakage of intracellular nucleic acids. Values are means ± SD (*p < 0.05, **p < 0.005, compared to DMSO or PBS-treated control).

To investigate the bacterial cell membrane damage by AP-AgNPs, fluorescent microscopy was conducted in the presence of propidium iodide as a marker of damaged membrane and acridine orange to stain all cells. Figure 6A displays the fluorescence images of MRSA after 24-h cultivation with AP-AgNPs at different concentrations. In the absence of AP-AgNPs, all cells had a green fluorescence representing an undamaged cytoplasmic membrane. In the presence of AP-AgNPs at ½ and 1× MIC red fluorescence was observed in approximately 30 and 60% of bacteria, respectively (Figures 6A,B). At 2× MIC, AP-AgNPs caused the loss of the membrane integrity by 92% of cells, respectively (Figures 6A,B).

Furthermore, PI staining using flow cytometry estimated cell membrane integrity. Generally, PI has no access to stain cellular DNA with an integral membrane. Fluorescence intensity signifies the number of impaired cells (Han et al., 2020). As shown in Figure 6C, MRSA treated with AP-AgNPs displayed a high percentage of stained cells by PI as compared to either control or vancomycin-treated cells. As shown in Figure 6D, after 30 min of treatment, the optical density values in the AP-AgNP group and the Triton X-100 group were higher as compared to the negative control group, demonstrating that AP-AgNPs increased the membrane permeability of MRSA. The results of intracellular protein and nucleic acids leakage are displayed in Figures 6E,F. AP-AgNPs exhibited a significant effect on MRSA cellular damage as revealed by releasing a high amount of protein and nucleic acids contents of MRSA cells as compared to control non-treated cells.

### 3.7 Effect of AP-AgNPs on MRSA biofilm formation

**Figure 7:**
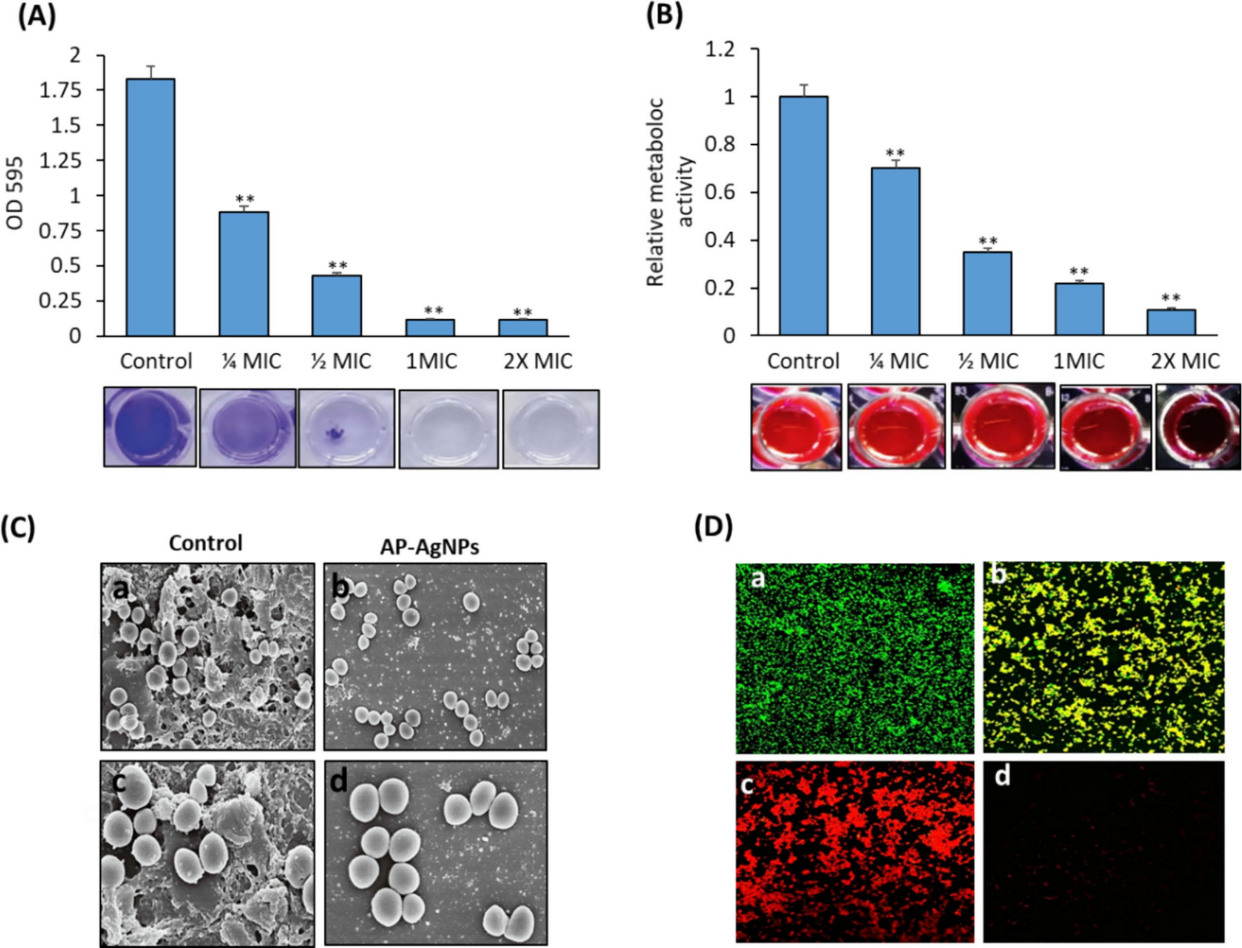
Effect of AP-AgNPs on biofilm formation by MRSA. (A) Crystal violet and (B) resazurin (excitation wavelength: 570 nm; emission wavelength: 600 nm). Values are means ± SD (**p < 0.005, compared to control cells). (C) Scanning electron microscopy images of biofilm formation of MRSA treated with (a,c) DMSO (control) or (b,d) AP-AgNPs at 35°C for 24 h. A significant bacterial biofilm growth, which led to clusters with composite morphology, was seen on the surface of the glass in the control group. However, limited bacterial biofilm formation was seen on the surface of the (AP-AgNPs) treatment group. (D) Confocal laser scanning microscopy image of LIVE/DEADH-stained demonstrating the effects of different AP-AgNP concentrations on MRSA biofilm formation. Biofilms were formed on cover slides within 48 h at 37°C. Biofilms were treated with AP-AgNPs for 48 h at 37°C. (a) Control (DMSO); (b–d) treatment with AP-AgNPs at 25 μg/mL, 50 μg/mL, or 100 μg/mL, respectively. Green, viable cells; Red, dead cells.

We examined the effect of AP-AgNPs on the suppression of MRSA biofilm formation by crystal violet staining to measure the mass of biofilm. As shown in Figure 7A, AP-AgNPs displayed a dose-dependent inhibitory effect on the biofilm medium, the formation of MRSA biofilm. Resazurin viability test was applied to measure the occurrence of active biofilm bacteria (Figure 7B). AP-AgNPs excreted an inhibitory effect on the metabolic activity of MRSA in a dose-dependent manner (Figure 7B). Furthermore, we explored the effects of AP-AgNPs on the adhesion of the MRSA biofilm on the glass surface using SEM. As shown in Figures 7C,a,c, control non-treated MRSA showed a significant bacterial biofilm growth, which led to clusters with complex morphology observed on the surface of the glass. In contrast, AP-AgNPs significantly inhibited the bacterial biofilm formation (Figures 7C,b,d). Furthermore, the effect of AP-AgNPs on established biofilms was investigated using confocal laser scanning microscopy (CLSM) (Figure 7D). After treatment for 48 h, the control group showed living bacterial cells (Figures 7D,a) while, treatment with 25 μg/mL (MIC) of AP-AgNPs reduced the bacterial population and decreased the number of bacteria in the biofilm (Figures 7D,b), and removed the biofilms. Furthermore, biofilm bacteria are killed by AP-AgNPs at concentrations of 50 μg/mL and 100 μg/mL, and these concentrations of AP-AgNPs were also able to separate biofilms (Figures 7D,c,d).

### 3.8 Antibacterial effect of AP-AgNPs against MRSA-induced superficial skin infection in mice

**Figure 8:**
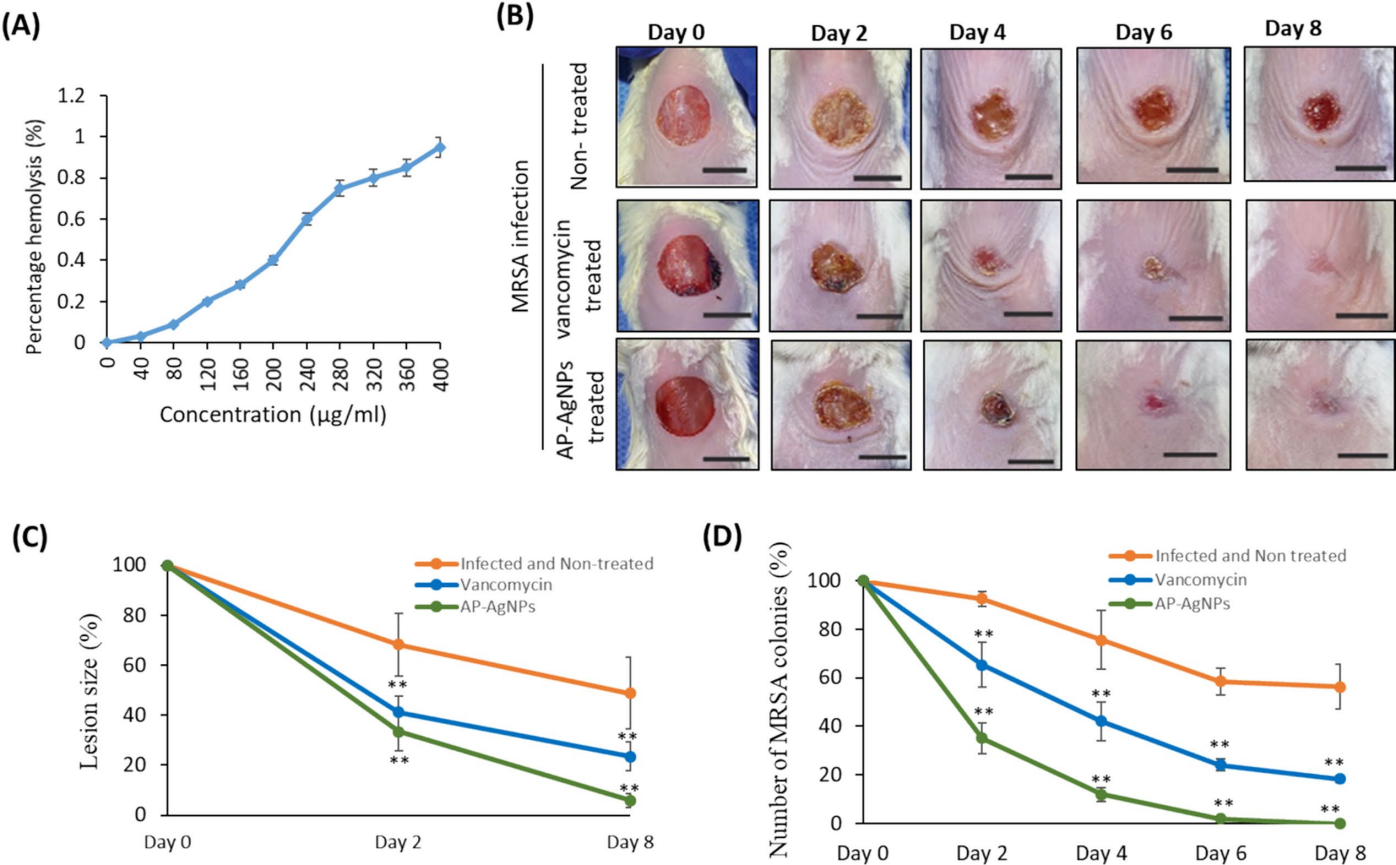
Mouse superficial wound infection. (A) Hemolytic activity of AP-AgNPs. The hemolytic activity of AP-AgNPs was estimated by monitoring the increase in the absorbance at 570 nm after incubating human red blood cells with different AP-AgNP concentrations at 37°C for 1 h. The positive control was 0.1% Triton X-100, and DMSO was used as a blank. (B) Mouse superficial wounds were performed by repeated tape stripping to remove the epidermis. Wounds were infected with 1 × 10^7^ MRSA colonies and treated every day with 10% ethanol in propylene glycol (non-treated control), 25 μg/mL AP-AgNPs (MIC) in 10% ethanol in propylene glycol or 50 μg/mL vancomycin (MIC) for eight days. The wounds of all mice were snapped on days 0–8. Scale bar: 1 cm. (C) Lesion size analysis on days 2 and 8 after initial surgery. (D) Numbers of MRSA colonies isolated from infected superficial mouse wounds under different treatments. Data are expressed as means ± SD (*n* = 6 mice/group) (**p < 0.005, compared to control infected and non-treated mice).

As a prerequisite step before applying AP-AgNPs in animals, the cytotoxicity of AP-AgNPs against mammalian cells was examined by a hemolysis test. The results confirmed that AP-AgNPs had lower hemolytic activity at a high concentration, and the HC_50_ value of AP-AgNPs was 340 μg/mL. At 200 μg/mL, the hemolytic activity of AP-AgNPs in human erythrocytes was lower than 30% (Figure 8A). At this concentration, AP-AgNPs efficiently suppressed the growth of MRSA cells.

To examine the potential wound healing potential of AP-AgNPs against MRSA-induced skin infection, we used a tape-stripping mouse model. In this model, infected mice with MRSA were treated topically with either saline, vancomycin, or AP-AgNPs. Topical treatment of MRSA-induced skin infection in mice with AP-AgNPs reduced the size of wounds significantly at days 2 and 8 as compared to control infected non-treated mice (Figures 8B,C). Precisely, on day 6, mice in the AP-AgNP and vancomycin groups showed fast control of the infection, as demonstrated by the absence of pus under the scab and greater wound healing; however, in the control group, pus persisted under the scab (Figures 8B,C). Conversely, scabs still strongly adhere to the non-treated tissues and approximately 30% of unhealed wounds continued noticeable.

To examine the *in vivo* anti-MRSA activity of AP-AgNPs, the bacterial burden in skin lesions was evaluated during the timeline of wound healing as described in the material and methods. Interestingly, treatment of MRSA-infected wounds with AP-AgNPs showed a significant reduction in the percentage of MRSA colonies that reached 0% after 6 days of treatment compared to vancomycin treatment mice (Figure 8D).

**Figure 9:**
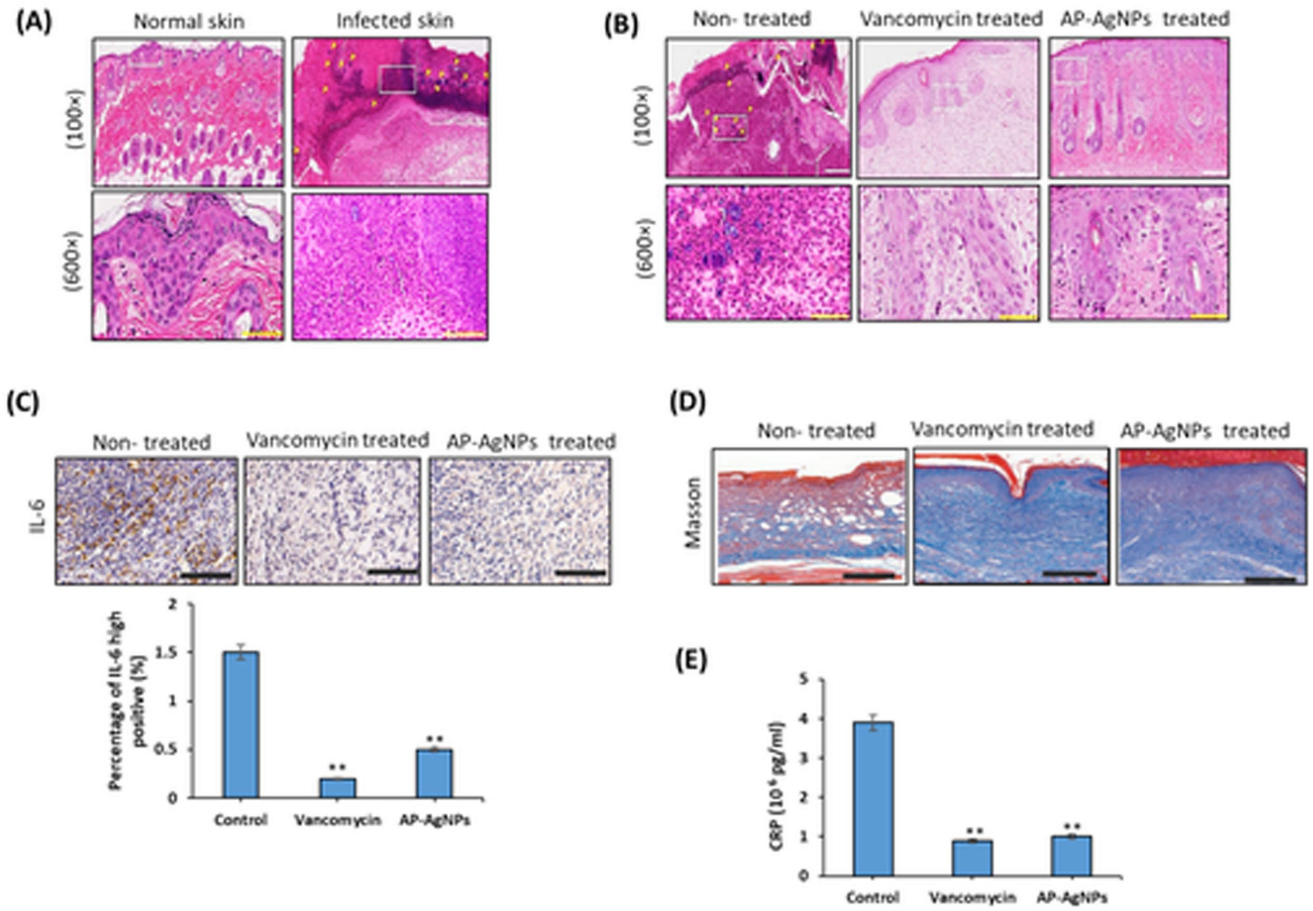
Histology of wounds with H&E staining. Mice were sacrificed and tissues were cut into 6-μm sections, stained with H&E, and mounted. (A) Skin sections comparing normal skin versus MRSA-infected skin after 2 days of infection. (B) MRSA-infected skin sections from mice non-treated or treated with vancomycin or AP-AgNPs after 8 days of treatment. Magnification = 100X and 600X. (C) Immunohistochemical staining for IL-6 in the hypodermis from the infected wounds on day 8; scale bar: 100 μm. (D) Masson’s staining of the tissues from the infected wounds on day 8; scale bar: 200 μm. (E) serum C-reactive protein (CRP) levels. Data are expressed as means ± SD (*n* = 6 mice/group) (**p < 0.005, compared to control infected and non-treated mice).

As shown in Figure 9A, compared to normal skin, abundant inflammatory cell infiltration (mostly neutrophil), great quantities of fragments of pus cells, necrotic tissues, and MRSA clusters were detected in the skin tissues after 2 days of infection by MRSA. On day 8, the healing effect of AP-AgNPs was equivalent to that of vancomycin, with totally healed wound tissues. No MRSA clusters or pus cell fragments were detected, and inflammatory cells were fewer disseminated or not detected (Figure 9B). Furthermore, a greater number of fibroblasts, substantial neovascularization, and even newborn hair follicle tissues were detected in the subcutaneous tissue just underneath the wound surface of AP-AgNP-treated groups, demonstrating good healing (Figure 9B). After 8 days, immunohistochemical (IHC) examination was performed to measure the expression of interleukin (IL-6), which is a significant inflammatory marker, in the hypodermis. The tissue areas displayed a substantial decrease in the infection-related inflammation of the wounds after treatment with AP-AgNPs on day 8 (Figure 9C). More thicker new shaped collagen fibers at the center of the wound area, were detected through Masson straining (Figure 9D) and serum C-reactive protein (CRP) levels were expressively decreased in the AP-AgNP group (Figure 9E). Our results proposed that AP-AgNPs could penetrate through the skin into the infected areas to eliminate MRSA.

## 4 Discussion

MRSA is the most common multidrug-resistant Gram-positive bacteria triggering healthcare-related infections. Consequently, MRSA is an essential goal for infection control and prevention measures (Xiao et al., 2024). This is the first report to provide the endophytic fungus *A. parasiticus-*based AP-AgNPs as an effective alternative treatment for MRSA-induced skin infection.

Fungi are considered the most suitable candidate for large-scale production of green nanoparticles. In this study, the mycosynthesized AP-AgNPs were established by an intense peak at 434 nm due to surface plasmon resonance that can be elucidated as the oscillation of free electrons of AgNPs with a specific energy, that scatters a specific wavelength of visible light (Ahmad et al., 2003). These data are in agreement with other reported mycosynthesized AgNPs (El-Zawawy et al., 2023; Gupta et al., 2022).

The crystalline nature of the AP-AgNPs was confirmed using the XRD pattern analysis, and the diffractogram was at the 2θ value of 38.19°, 44.37°, 64.56°, and 77.47°. This is due to the extracellular enzymes and proteins produced from *A. parasiticus*. These enzymes during the electron transfer process decrease the silver ions that give a brown color to the solution. Several other observations have revealed the participation of NADH and NADH-dependent nitrate reductase in the biosynthesis of AgNPs. The role of nitrate reductase activity was reported in the biosynthesis of AgNPs mediated by *Aspergillus flavus* (Zomorodian et al., 2016). The FTIR spectrum of AP-AgNPs designated the occurrence of alcoholic and phenolic compounds as well as proteins that might be attributed to biomolecules involved in the reduction in silver ions and stabilization of particles (Ebrahiminezhad et al., 2017).

Our results established the spherical-oblate shape of AP-AgNPs, with an average particle size of 27.7 nm. The AgNPs biosynthesized using the endophytic fungus *Aspergillus versicolor* isolated from the *Centella asiatica* plant was spherical in shape and well dispersed with sizes ranging from 3 to 40 nm (Netala et al., 2016). Zeta potential assessment is an important and easiest method of calculating the stability and understanding of nanoparticle surface quality. Zeta potential for the reaction mixture of AP-AgNPs showed a negative value (−25.5 ± 3.15 mV). Consistent with this, the zeta potential of silver nanoparticles produced by other endophytes such as *Aspergillus versicolor* isolated from the *Centella asiatica* and *Talaromyces purpureogenus* isolated from *Taxus baccata* was equal to −38.2 mV, − 19.5 mV, respectively (Netala et al., 2016; Sharma et al., 2022). The verification of the presence of silver was performed by EDX analysis. The optical absorption peak of AP-AgNPs appears at approximately 3 KeV, which is similar to that of metallic silver nanoparticles because of SPR (Park et al., 2007). Other EDX signals released from O, N, and C are possible because of X-ray emission from proteins existing in the cell-free filtrates, that could bind to nanoparticles either via free amino groups or cysteine residues (Mandal et al., 2005).

The mechanism of mycosynthesis of AgNPs by fungi was proposed to be due to the presence of biomolecules such as proteins, amino acids, enzymes, vitamins, and polysaccharides in the supernatant of fungi that might act as bioreductant and capping agents (Gudikandula et al., 2017). In addition, the presence of the enzyme nicotinamide adenine dinucleotide (NADH) and NADH-dependent nitrate reductase was reported to play an essential role in this process (Kalimuthu et al., 2008).

Our results revealed that the MIC of AP-AgNPs was 25 μg/mL. The MIC values are greatly dependent on the method of nanoparticle synthesis, their physicochemical characteristics, methods for MIC assessments, and the density of the target bacterial inoculum (Budzyńska et al., 2017). It is worth noting that because of an absence of calibration, the anti-MRSA activity of AgNPs recognized in publications is unreliable. For example, AgNPs biosynthesized from starch exhibited powerful antibacterial activity against MRSA with MIC value 8.125 μg/mL (Salah et al., 2021), while chemically synthesized AgNPs showed higher anti-MRSA activity with MIC ranging from 11.25 to 45 μg/mL (Ansari et al., 2015).

The antibacterial activity of AP-AgNPs against MRSA could be due to the function of AP-AgNPs as a carrier of fungal enzymes and proteins, which are used as capping and stabilizing agents, assisting the penetration into bacterial cells. Additionally, AgNPs directly adhere to the bacterial surface, changing the structural integrity of the membrane. Then, AgNPs penetrate the bacterial cell and interact with its intracellular components, destroying it until it cannot do dynamic cellular processes (Lee and Jun, 2019). Moreover, AgNPs are capable of inducing reactive oxygen species and generating free radicals, therefore triggering irreversible oxidative injury to the bacteria (Durán et al., 2016; Yan et al., 2018). Modification of vital signaling transduction, which is essential for the bacterial life cycle, is also one of the modes of action displayed by AgNPs (Masimen et al., 2022). In this context, fungal extracts showed to be valued source for several secondary metabolites that act as an antibacterial agents. Interestingly, our GC–MS analysis of *A. parasiticus* extract revealed the presence of a high percentage of fungal secondary metabolites with an antibacterial activity that was capped by AgNPs and enhanced their anti-MRSA activity. These fungal secondary metabolites include 9-octadecenoic acid (Z)-, methyl ester (El-Fayoumy et al., 2021), 9-octadecenoic acid (E)- (Mohy El-Din and Mohyeldin, 2018), 10,13-octadecadiynoic acid, methyl ester (Agoramoorthy et al., 2007), androst-4-en-3-one, 17-methoxy-,3-methoxime, (17á)- (Amiranashvili et al., 2020), and 1,2-benzenedicarboxylic acid, diisooctyl ester (Gopalasamy et al., 2013).

Treatment of MRSA with AP-AgNPs was shown to increase their cellular membrane permeability and leakage of cellular components. This is due to the mechanical consequence of caveating bubbles formed by AP-AgNPs, which significantly internalize AP-AgNPs into MRSA cells and stabilize their interaction with cellular components such as protein, DNA, and lipids (López-Heras et al., 2015).

In this study, SEM and TEM micrographs demonstrated that the AP-AgNP treatment has rendered cell membrane ruptures, cell wall injury, empty cell envelopes, and cell fragments. The distinctive deformities in the MRSA cell structure treated by AP-AgNPs propose the production of defective cell wall material that resulted in the formation of an aberrant situation. Therefore, AP-AgNPs affect either the cell wall metabolism or the membrane components. It was reported, that the interactions between AgNPs and MRSA cell wall resulted in cytoplasmic leakage, distortion of cell shape bacterial cell wall, damage of the peptidoglycan layer, porosity of the cell membrane, and subsequent leakage of cytoplasmic material, which finally causes bacterial cell lysis (Romero-Urbina et al., 2015). In addition, binding of AgNPs to bacterial membranes resulted in deformed cell surface, holes, depressions, and cell leakage, which might consequently be from the disturbed cell permeability and the consequent injury caused by silver ions or nanoparticles to the cell structure (Agrawal et al., 2021).

Bacterial biofilms are a major reason for disease and drug resistance. 3D structures of bacterial biofilms are formed by surrounding microbial groups in a matrix of self-produced extracellular polymeric substances (Bridier and Briandet, 2022). Our results revealed that the formation of MRSA biofilm was powerfully repressed with increasing concentration of AP-AgNPs. Furthermore, the metabolic activity of MRSA cells was considerably reduced by AP-AgNPs in pre-grown structures as compared to the control group. Additionally, the results of SEM revealed limited bacterial biofilm formation on the surface of MRSA treated with AP-AgNPs. Interestingly, AP-AgNPs have a destroying effect on established MRSA biofilms as detected using confocal laser scanning microscopy (CLSM). These results designate that AP-AgNPs could efficiently inhibit the formation of MRSA biofilms. In this context, AgNPs biosynthesized using dried orange peel extract showed inhibitory effects against biofilm formation by MRSA at a concentration of 1/8 × MIC (0,195 μg/mL) (Merghni et al., 2022). Biosynthesized AgNPs using cell-free enzyme of *Fusarium oxysporum* inhibited biofilm formation at 4× to 1/8 MIC (11.7 to 750 μM) concentrations for all tested MRSA strains when compared with the control group (da Silva et al., 2024). Similarly, Hu et al. (2023) revealed that confocal laser scanning microscopy (CLSM) images of the live−/dead-stained bacteria displayed that remarkably more red bacterial cells were observed after the treatment with T ^2^A^2^ (two aldehyde molecules reacted with the primary amine of DN). Additionally, T ^2^A^2^ could accomplish the synergic effect of NO and natural aldehydes in eliminating either drug-resistant bacteria or their biofilms.

In general, nanoparticles could interact with bacterial biofilm in three steps. The first interaction concerns the transport of these particles around the biofilm. The second one is their attachment to the surface of bacterial biofilm and finally the migration within this structure (Ikuma et al., 2015). However, the precise mechanism by which AgNPs suppress biofilm formation is unclear. It was suggested that anti-biofilm activity is attributed to the superficial binding of AgNP and increases penetration into the biofilm, disturbing the lipidome of cell membranes (Ahamad et al., 2021). Additionally, AgNPs are reported to induce the production of reactive oxygen species (ROS), causing oxidative stress (Wang et al., 2017). ROS could cause harmful modifications to cellular constituents and damage proteins, DNA, and lipids (Su et al., 2019).

Several *in vivo* skin animal models were established to investigate the MRSA virulence. These include the tape-stripping mouse model (Tatiya-Aphiradee et al., 2016), the murine skin infection model (Zhang et al., 2021), the mouse subcutaneous infection model (Abtin et al., 2014), and an experimental mouse model of foreign body infection (Espersen et al., 1994). In this study, we used the superficial skin infection using the tape-stripping mouse model to investigate the antibacterial effect of AP-AgNPs against MRSA. This is an easy, biologically relevant, and re-producible *in vivo* model for identifying effective therapeutic agents for infected wound healing (Kugelberg et al., 2005; Tatiya-Aphiradee et al., 2019).

Topical treatment of MRSA-infected wounds with AP-AgNPs showed complete regeneration of the epidermis layer with no inflammatory cell infiltration and clearance of MRSA colonies, suggesting the effective therapeutic potential of AP-AgNPs against MRSA *in vivo*. Plant extracts as a source of phytochemicals with antibacterial activity were used in a few studies to biosynthesis AgNPs that are effectively used against MRSA (Fatima et al., 2023; Kasithevar et al., 2017). In addition, the wound-healing potential of AgNPs was reported in animal models using green synthesized AgNPs (Jain et al., 2009). On the other hand, endophytic fungi isolated from medicinal and wild plants such as *Ageratina adenophora* (Wen et al., 2023), *Coptis chinensis* (Ming et al., 2022), and *Tradescantia pallida* (Dhevi et al., 2024) demonstrated effective antibacterial activity against MRSA. However, there are no studies that used entophytic fungi-based AgNPs against MRSA. To date, this is the first report to use endophytic fungi-based AgNPs as a novel antibacterial agent against MRSA-induced skin infection. Our data provide an effective anti-MRSA green drug, which combines the characteristic antibacterial activities of both phytochemicals from *Reseda arabica* and endophytes from *A. parasiticus* that act as capping and stabilizing agents for AgNPs.

## 5 Conclusion

In this study, we provided AP-AgNPs biosynthesized with cell-free filtrate of endophytic fungus *A. parasiticus* (AP-AgNPs) as an alternative antibacterial treatment for MRSA. Our results showed that AP-AgNPs inhibited growth, the development of bacterial biofilms, and degenerated bacterial membranes and cell walls by inhibiting ROS production. Interestingly, the topical application of AP-AgNPs is highly effective against cutaneous MRSA infection in a murine model. Additionally, the AP-AgNPs seem to improve wound healing. Therefore, our data demonstrated AP-AgNPs as a hopeful antibacterial drug for the treatment and/or prevention of MRSA via inhibiting the key virulence factors.

## Data Availability

The raw data supporting the conclusions of this article will be made available by the authors, without undue reservation.

## Footnotes

^1^http://rsb.info.nih.gov/ij
