# Supplementary material for: Mycosynthesis of silver nanoparticles from endophytic Aspergillus parasiticus and their antibacterial activity against methicillin-resistant Staphylococcus aureus in vitro and in vivo
Source: Front Microbiol. 2024 Nov 15;15:1483637. doi: 10.3389/fmicb.2024.1483637 (PMC11604631; doi:10.3389/fmicb.2024.1483637)
Supplement: Supplementary file 1 [file Data_Sheet_1.pdf]

Supplementary figure 1

(A)

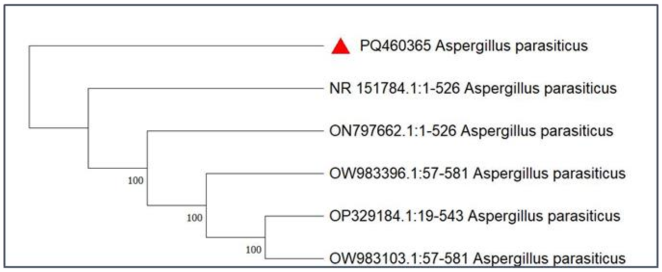

(B)

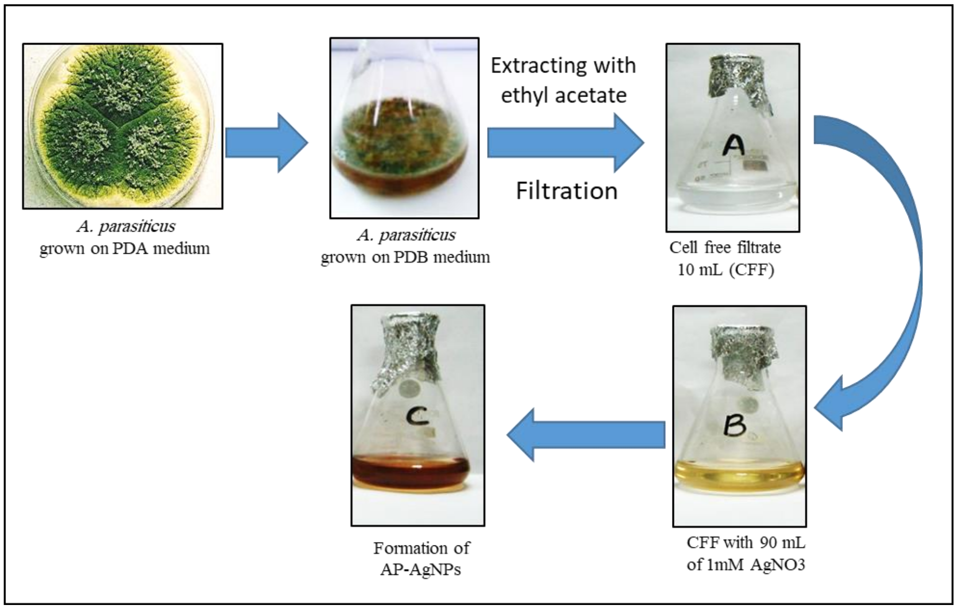

Figure S1. (A) Phylogenetic tree based on the 5.8S-rRNA-ITS regions between the endophytic fungus *A. parasiticus* and published data and evolutionary distances computed using the Maximum Likelihood method. (B) Biosynthesis of silver nanoparticles. Color changes from light yellow to dark brown following the incubation at room temperature indicates the reduction of the Ag+ ions and the biosynthesis of AgNPs.
